# An Adaptive Calibration Algorithm Based on RSSI and LDPLM for Indoor Ranging and Positioning

**DOI:** 10.3390/s22155689

**Published:** 2022-07-29

**Authors:** Jingmin Yang, Shanghui Deng, Minmin Lin, Li Xu

**Affiliations:** 1 School of Computer Science, Minnan Normal University, Zhangzhou 363000, China; dsh0106@126.com (S.D.); 18876315731@163.com (M.L.); 2 Key Laboratory of Data Science and Intelligence Application, Minnan Normal University, Zhangzhou 363000, China; 3 Fujian Provincial Key Laboratory of Network Security and Cryptology, Fujian Normal University, Fuzhou 350007, China; xuli@fjnu.edu.cn

**Keywords:** adaptive calibration, error correction linear regression equation, indoor ranging and positioning, logarithmic distance path loss model, received signal strength indication

## Abstract

The positioning algorithm based on received signal strength indication (RSSI) and the logarithmic distance path loss model (LDPLM) is widely used in indoor positioning scenarios due to its convenient detection and low costs. However, the classic LDPLM with fixed coefficients and fixed error estimation usually reduces the ranging accuracy, but it is rarely studied in previous literature. This study proposes an adaptive calibration ranging algorithm based on LDPLM, which consists of two parts: coefficient adaptive algorithm and error correction algorithm. The coefficient adaptive algorithm is derived by utilizing the error theory and the least squares method. The error correction algorithm is defined as the linear regression equation, in which coefficients are determined by the least squares method. In addition, to reduce the influence of RSSI’s fluctuation on ranging accuracy, we propose a simple but effective filtering algorithm based on Gaussian. The experimental results show that compared with the classic LDPLM and polynomial fitting model, the ranging accuracy of the proposed algorithm is improved by 58% and 51%, respectively, and the positioning cumulative prediction error of the proposed model is reduced by 69% and 80%, respectively.

## 1. Introduction

Location-based services (LBS) is an information service model that integrates multiple location technology and data processing technology, and uses modern communication technology to provide end users with comprehensive location information-related services [1]. With the rapid development of wireless communication networks and mobile Internet, LBS has become a business with broad market prospects. For example, in the consumer field, LBS is widely used in indoor navigation and information-pushing service. In the field of safety emergency response, LBS is applied to underground positioning of the coal mine, which can provide early warning before the disaster occurs, quickly locate the miners, and guide the robot to rescue when a disaster occurs. In addition, in the sectors of education, tourism, medical care, and others, LBS also has a wide range of application needs [2]. Positioning technology is an important prerequisite for LBS. Therefore, it is of great significance to study the positioning algorithm with high positioning accuracy and fast positioning speed.

In general, positioning is divided into indoor and outdoor scenarios. In indoor environment, positioning using the wireless local area network (WLAN) as the basic infrastructure is gaining increasing attention. WLAN is based on the IEEE 802.11 series standards issued by the Institute of Electrical and Electronics Engineers (IEEE). It uses high-frequency radio frequency signals, such as wireless electromagnetic waves in the 2.4 GHz or 5 GHz band, as transmission medium. In 2018, a textbook titled “Wireless network technology: principle, experiment, and network design” states that WLAN has the characteristics of flexible use, convenient expansion, effective cost, and simple installation [3]. Meanwhile, with the advancement of wireless communication technologies and the development of indoor communication requirements, WLAN has developed rapidly. According to a report titled “Cisco Annual Internet Report (2018–2023)” released by Cisco Systems Inc., by 2023, the number of global public WLAN hotspots will increase from 169 million in 2018 to nearly 628 million, a fourfold increase [4]. WLAN has been widely constructed as the fundamental infrastructure of indoor wireless communication, as well as a large number of smartphones and personal computers supporting WLAN have been popularized, which provides favorable conditions for utilizing existing WLAN infrastructure for indoor positioning.

In recent years, many scholars have carried out a lot of research on the indoor positioning technology based on WLAN, and have achieved fruitful results [5]. For example, the ranging positioning technology is a typical kind of achievement [6]. The principle of ranging positioning technology is to measure the distance, time difference, or angle information between anchor points firstly, and then calculate the specific coordinates of to-be-positioned points by utilizing positioning algorithms such as trilateral measurement, triangulation, or maximum likelihood estimation. Typical ranging techniques include time of arrival (ToA), time difference of arrival (TDoA) [7,8], angle of arrival (AoA) [9,10], frequency difference of arrival (FDoA) [11,12], and RSSI [13,14]. The LDPLM model is a classic RSSI-based ranging algorithm, which realizes ranging by establishing a mapping relationship between RSSI and distance. Compared with the previous ranging techniques, LDPLM is more widely used due to its advantages of easy deployment, low power consumption, and low costs. However, the classic LDPLM has two main defects, which affect the ranging accuracy. One is the estimation of the coefficient term and error term of the model. To solve this problem, some scholars propose improved algorithms to estimate the coefficient and error term of the LDPLM. For example, Li Guoquan et al. used back propagation neural network optimized by particle swarm optimization to train the LDPLM model to reduce the positioning error [15]. Liu Yong et al. proposed the FA and PSO algorithm to optimize the parameters of the model, and then correct the ranging error [16]. The other is the fluctuation of RSSI. In fact, all kinds of RSSI-based ranging algorithms encounter this problem. The reason is that the transmission of WLAN signal is easily affected by the indoor environment, resulting in fluctuations. In 2020, we conducted systematic and quantitative research on the factors affecting the indoor accuracy of RSSI-based positioning. Our research notes that, in addition to the multi-path effect, the RSSI signal is also greatly affected by factors such as co-frequency interference and adjacent frequency interference, direction and height differences between the receiver antenna and the transmitter antenna, and the hardware heterogeneity of receiving devices [17].

To improve the ranging accuracy and reduce the positioning prediction error of the classic LDPLM, we propose the adaptive calibration algorithm based on LDPLM for indoor ranging and positioning. The adaptive calibration algorithm consists of two parts: coefficient adaptive algorithm and error correction algorithm. In this paper, the term anchor point refers to the coordinates of the point where the access point (AP) is located. The term reference point refers to the point with known coordinates of a specific location. Both the anchor point and the reference point are used to solve the undetermined parameters of the adaptive calibration algorithm. The term to-be-positioned point refers to the point where the position coordinates need to be estimated. The main contributions of our work are as follows.

(1) We design a coefficient adaptive algorithm, which can dynamically calculate the coefficients of the classic LDPLM according to the RSSI at different anchor points and reference points. Furthermore, we design an error correction linear regression equation to reduce the error caused by the coefficient adaptive algorithm. Compared with the classic LDPLM model and the polynomial fitting ranging model, the adaptive calibration algorithm can effectively improve the ranging accuracy and reduce the positioning prediction error and positioning cumulative prediction error.

(2) Based on our previous research on indoor RSSI interference factors, we design a simple but effective filtering algorithm based on Gaussian. The filtering algorithm adopts the 2σ criterion to process RSSI, which is used as the input of the proposed adaptive calibration algorithm.

The rest of this paper is organized in the following way. Section 2 introduces the related background theory and technology. Section 3 presents the proposed methodology in detail. Section 4 illustrates the experimental setup and analyzes the experiment results. Finally, conclusions and suggestions for future works are discussed in Section 5.

## 2. Preliminaries

In this section, we introduce the related background theory and technology, mainly including the classic LDPLM and polynomial fitting model used for comparative experiments, the least squares method for solving positioning coordinates, and the RSSI Gaussian filtering algorithm.

### 2.1. Classic LDPLM

The LDPLM points out that the range fading characteristics of WLAN signal in an indoor environment obey the lognormal distribution [18]. In free space, assume that *F* is the electric field power density of the transceiver antenna, $G_{r}$ is the gain of the transceiver antenna, λ is the wavelength of the WLAN signal, and *L* is the system loss. Therefore, the received signal power $P_{r}$ can be expressed as

(1) $$P_{r}=\frac{FG_{r}\lambda^{2}}{4\pi L}$$

When the wireless signal propagates in space, the signal power will attenuate with distance, so the electric field power density of the transceiver antenna *F* can be expressed as

(2) $$F=\frac{P_{t}G_{t}}{4\pi d^{2}}$$

where $P_{t}$ is the transmitter signal power, and $G_{t}$ is the antenna gains of the transmitter and the transceiver. In this paper, the transmitter refers to the AP of WLAN, and the transceiver refers to the wireless network interface card (WNIC) supporting WLAN. Parameter *d* refers to the distance between the transmitter and the transceiver. Bring Formula (2) into Formula (1) and ignore the system loss *L*, the power received by the transceiver at a distance *d* from the transmitter obeys the Fries transfer formula. Therefore, this mapping relationship can be expressed as follows:

(3) $$P_{r}\left(d\right)={\left(\frac{\lambda }{4\pi d}\right)}^{2}P_{t}G_{t}G_{r}$$

Assume that the antenna gains of the transmitter and the transceiver are the same, as well as considering the fading effect of WLAN signals transmission in the indoor environment, the expression (3) can be embodied into classic LDPM, as shown below:

(4) $$R\left(d\right)=R_{0}\left(d_{0}\right)-10nlog\left(\frac{d}{d_{0}}\right)+X_{\sigma }$$

where R(d) and $R_{0}\left(d_{0}\right)$ represent the RSSI of the reference point at the distance *d* and $d_{0}$, respectively. *n* is the path loss coefficient related to the specific indoor environment, which represents the change speed of the path loss with the increase of distance. $X_{\sigma }$ is the noise interference represented by a Gaussian normally distributed random variable with a mean value of 0 and mean square error value of σ, which is used to indicate the error of the model.

In the classic LDPLM, R(d) uses the mean value of RSSI measured many times at the to-be-positioned point, $R_{0}\left(d_{0}\right)$ uses the mean value of RSSI measured many times at the reference point with 1 m, while *n* and $X_{\sigma }$ usually take a fixed value based on experience. Adopting fixed coefficients leads to the relatively large error of calculated distance *d*. In Section 3, we propose a coefficient adaptive algorithm, which dynamically calculates the corresponding coefficient term *n* according to the input RSSI of different anchor points and reference points.

### 2.2. Polynomial Fitting Ranging Model

The polynomial fitting ranging model mainly establishes a polynomial fitting function between the RSSI and the distance according to the physical characteristics of the RSSI attenuating with the increase of distance in indoor environment [18].

First, measure the actual measurement distance $d_{i}$, *i* = 1, 2, …, *m*, from *m* points with known specific coordinate points, i.e., reference points, to a certain transmitter and the actual measurement mean value $\bar{R(d_{i})}$ of the transmitter RSSI continuously measured at *m* points with known specific coordinate points. Then, establish the polynomial fitting ranging model of $\bar{R(d_{i})}$ and $d_{i}$, as shown in the following expression:

(5) $$\left\{\begin{array}{c} \bar{R(d_{1})}=a+bd_{1}+cd_{1}^{2}\\ \bar{R(d_{2})}=a+bd_{2}+cd_{2}^{2}\\ \vdots \\ \bar{R(d_{m})}=a+bd_{m}+cd_{m}^{2} \end{array}\right.$$

Formula (5) is for a single transmitter, in which coefficients a,b,c are to be determined. In order to determine the coefficients of a,b and *c* for a specific transmitter, it is only necessary to collect the data of $\bar{R(d_{i})}$ and $d_{i}$ from three reference points. The polynomial fitting ranging model is a simple but effective model. Yao Jinyi et al. pointed out in a study that in fitting models of RSSI and distance, the polynomial fitting ranging model has higher ranging accuracy than the linear or the logarithmic fitting ranging models [18].

### 2.3. Positioning Based on Least Squares

The least squares method is an effective mathematical optimization technology. It finds the best fitting function of test data by minimizing the sum of squares of errors. Gauss-Markov theory points out that under the assumption of classical linear regression, the least squares estimator is a linear unbiased estimator with minimum variance. In other words, compared with any linear unbiased estimator obtained by other methods, the least square method is the best.

Assume that there are m(m⩾3) APs in the indoor environment, and their known coordinates are $\left(x_{1},y_{1}\right),\left(x_{2},y_{2}\right),\ldots ,\left(x_{m},y_{m}\right)$, respectively. The coordinate of to-be-positioned point *A* is A(x,y). The distances from to-be-positioned point *A* to each AP are $d_{1},d_{2},\ldots ,d_{m}$. Thus, simultaneous equations are given as follows:

(6) $$\left\{\begin{array}{c} {\left(x-x_{1}\right)}^{2}+{\left(y-y_{1}\right)}^{2}=d_{1}^{2}\\ \vdots \\ {\left(x-x_{m}\right)}^{2}+{\left(y-y_{m}\right)}^{2}=d_{m}^{2} \end{array}\right.$$

The simultaneous equations are a system of nonlinear equations. To solve this system of nonlinear equations, we subtract the *m*-th equation from the previous n−1 equations to obtain a linearized equation

(7) AX=b

where

$A=\left(\begin{array}{cc} 2(x_{1}-x_{m}) & 2(y_{1}-y_{m})\\ 2(x_{2}-x_{m}) & 2(y_{2}-y_{m})\\ \vdots & \vdots \\ 2(x_{m-1}-x_{m}) & 2(y_{m-1}-y_{m}) \end{array}\right)$

$b=\left(\begin{array}{c} x_{1}^{2}-x_{m}^{2}+y_{1}^{2}-y_{m}^{2}+d_{m}^{2}-d_{1}^{2}\\ x_{2}^{2}-x_{m}^{2}+y_{2}^{2}-y_{m}^{2}+d_{m}^{2}-d_{2}^{2}\\ \vdots \\ x_{m-1}^{2}-x_{m}^{2}+y_{m-1}^{2}-y_{m}^{2}+d_{m}^{2}-d_{m-1}^{2} \end{array}\right)$, $X=\left(\begin{array}{c} x\\ y \end{array}\right)$

The least square method is used to find the optimal solution of the Equation (7). the error ε existing in the measurement can be expressed as follows:

(8) $$Q\left(x\right)={∥\varepsilon ∥}^{2}={∥AX-b∥}^{2}$$

To obtain the minimum value of Q(x), the differential derivation of Equation (8) is as follows:

(9) $$\frac{dQ(x)}{dx}=2AA^{T}x-2Ab=0$$

If $AA^{T}$ is nonsingular, the solution *X* is as follows:

(10) $$X={\left(AA^{T}\right)}^{-1}Ab$$

### 2.4. RSSI Gaussian Filtering Algorithm

Our previous research points out that RSSI is greatly affected by the indoor environment, which affects the ranging accuracy of RSSI-based ranging algorithms [17]. Therefore, before actually using RSSI values, it is necessary to eliminate abnormal values from the RSSI collected in the experiment to obtain a set of relatively smooth values. A large number of studies show that in the indoor environment, the random variable RSSI approximately obeys the normal distribution in most cases [19]. Therefore, the 3σ criterion is used to discriminate errors and eliminate outliers is adopted in this paper.

The 3σ criterion assumes that the test data contains only random errors. The original data is processed to obtain the standard deviation, and then an interval is determined according to a certain probability. Values exceeding this interval are considered outliers. Reference [20] assumes that the RSSI in the indoor environment obeys a Gaussian distribution with mean value μ and variance $\sigma^{2}$. Then its probability density function can be expressed as follows:

(11) $$f\left(RSSI\right)=\frac{1}{\sigma \sqrt{2\pi }}exp\left(-\frac{{\left(RSSI-\mu \right)}^{2}}{2\sigma^{2}}\right)$$

where $\mu =\frac{1}{n}\sum_{i=1}^{n}\left(RSSI\left(i\right)\right)$, $\sigma =\sqrt{\frac{1}{n-1}\sum_{i=1}^{n}{\left(RSSI\left(i\right)-\mu \right)}^{2}}$.

This paper selects the 2σ as the range to remove outliers from the collected data. Specifically, calculate the arithmetic mean of all RSSI values in the range of [μ−2σ,μ−2σ] as the final RSSI value for ranging.

## 3. Introduction to the Adaptive Calibration Algorithm

This section is divided into two parts: the first one introduces the adaptive calibration algorithm, and the second part describes the positioning process. The adaptive calibration algorithm includes coefficient adaptive algorithm and error correction algorithm. The coefficient adaptive algorithm is used for dynamically calculating the coefficients of the classic LDPLM. The error correction algorithm is used for reducing the error caused by the coefficient adaptive algorithm.

### 3.1. Coefficient Adaptive Algorithm

In the classic LDPLM, as shown in the expression (4), assume that the coordinate of the anchor point *A* and the *m* reference points are known, and the distances from the *m* reference points to the anchor point *A* are also known as $d_{1},d_{2},\ldots ,d_{m}$. An AP is placed at the anchor point *A*. Then, The RSSI of the AP at the anchor point *A* is continuously measured for a period of time at each reference point, and the average value $\bar{R(d_{i})}$ is calculated after processing by the 2σ criterion described in Section 2. Thus, the above process can be expressed as follows:

(12) $$\left\{\begin{array}{c} \bar{R(d_{1})}=R_{0}\left(d_{0}\right)-10nlg\left(\frac{d_{1}}{d_{0}}\right)+X_{\sigma 1}\\ \bar{R(d_{2})}=R_{0}\left(d_{0}\right)-10nlg\left(\frac{d_{2}}{d_{0}}\right)+X_{\sigma 2}\\ \vdots \\ \bar{R(d_{m})}=R_{0}\left(d_{0}\right)-10nlg\left(\frac{d_{m}}{d_{0}}\right)+X_{\sigma m} \end{array}\right.$$

According to the principle of indirect adjustment and error theory, the error equation of the form $V=B\hat{x}-L$ can be obtained as follows by transforming Expression (12):

(13) $$\left(\begin{array}{c} v_{1}\\ v_{2}\\ \vdots \\ v_{m} \end{array}\right)=\left(\begin{array}{cc} 1 & -10lg(\frac{d_{1}}{d_{0}})\\ 1 & -10lg(\frac{d_{2}}{d_{0}})\\ \vdots & \vdots \\ 1 & -10lg(\frac{d_{m}}{d_{0}}) \end{array}\right)\left(\begin{array}{c} R_{0}\left(d_{0}\right)\\ n \end{array}\right)-\left(\begin{array}{c} \bar{R(d_{1})}-X_{\sigma 1}\\ \bar{R(d_{2})}-X_{\sigma 2}\\ \vdots \\ \bar{R(d_{m})}-X_{\sigma m} \end{array}\right)$$

where $V={\left(v_{1},v_{2},\ldots ,v_{m}\right)}^{T}$, $B=\left(\begin{array}{cc} 1 & -10lg(\frac{d_{1}}{d_{0}})\\ 1 & -10lg(\frac{d_{2}}{d_{0}})\\ \vdots & \vdots \\ 1 & -10lg(\frac{d_{m}}{d_{0}}) \end{array}\right)$ $,L=\left(\begin{array}{c} \bar{R(d_{1})}-X_{\sigma 1}\\ \bar{R(d_{2})}-X_{\sigma 2}\\ \vdots \\ \bar{R(d_{m})}-X_{\sigma m} \end{array}\right)$.

According to the principle of the Least squares method, assume that each measurement is an independent equal precision observation, the coefficients in expression (13) can be determined by minimizing the sum of square error between the predicted value of the RSSI calculated by the model $\bar{R(d_{i})}$ and the actual value of the RSSI $R(d_{i})$, that is

(14) $$minV^{T}V=min\sum_{i=1}^{m}v_{i}^{2}=min\sum_{i=1}^{m}\left(B^{T}B{\hat{x}}^{T}x-2BL\hat{x}+L^{T}L\right)$$

In expression (14), take the partial derivative with respect to $\hat{x}$, we can get:

(15) $$B^{T}B\hat{x}-B^{T}L=0$$

(16) $$\hat{x}=\left(\begin{array}{c} R_{0}\left(d_{0}\right)\\ n \end{array}\right)={\left(B^{T}B\right)}^{-1}B^{T}L$$

Through the above method, the model coefficients can be dynamically and independently determined for the AP at each anchor point. Next, we present a linear regression equation to reduce the noise interference $X_{\sigma }$ in detail.

### 3.2. Error Correction Algorithm

Due to the influence of an indoor complex environment, there are errors between the measured value of RSSI and the real value of RSSI. In expression (12), these errors are expressed as the noise interference $X_{\sigma }$. Based on the expression (12), a linear regression equation is defined to correct the errors. The new model is constructed as follows:

(17) $$\left\{\begin{array}{c} {d_{i}}^{\prime }=10^{\frac{R_{0}\left(d_{0}\right)-\bar{R(d_{i})}+X_{\sigma i}}{10n}}\cdot d_{0}\\ {d_{i}}^{\prime \prime }=a+b{d_{i}}^{\prime } \end{array}\right.$$

In expression (17), Equation (1) is transformed from expression (12) to calculate the distance ${d_{i}}^{\prime }$ from the reference point *i* to the anchor point. On this basis, a distance error correction linear regression equation ${d_{i}}^{\prime \prime }=a+b{d_{i}}^{\prime }$ is defined to adjust the distance ${d_{i}}^{\prime }$ calculated by Equation (1), where *a* and *b* are linear regression coefficients.

In the actual measurement, we set a set of reference points for each anchor point, and the distance from each reference point to this specific anchor point is known as $\left(d_{1},d_{2},\ldots ,d_{m}\right)$. To determine the linear regression coefficients *a* and *b*, we use the method that minimizes the sum of the squared differences of the true and calculated distances, that is $min\sum_{i=1}^{m}{\left({d_{i}}^{\prime }-d_{i}\right)}^{2}$.

In order to eliminate the error among multiple measurements, according to the principle of indirect adjustment and error theory, the error equation of the form $V=C\hat{x}-L$ can be obtained as follows:

(18) v1v2⋮vm=1d1′1d2′⋮⋮1dm′ab−d1d2⋮dm

where $V={\left(v_{1},v_{2},\ldots ,v_{m}\right)}^{T}$, $C=\left(\begin{array}{cc} 1 & {d_{1}}^{\prime }\\ 1 & {d_{2}}^{\prime }\\ \vdots & \vdots \\ 1 & {d_{m}}^{\prime } \end{array}\right)$, $L=\left(\begin{array}{c} d_{1}\\ d_{2}\\ \vdots \\ d_{m} \end{array}\right)$.

According to the least square principle, the linear regression coefficients can be determined as:

(19) ab=(CTC)−1CTL

### 3.3. Localization Process

**Figure 1:**
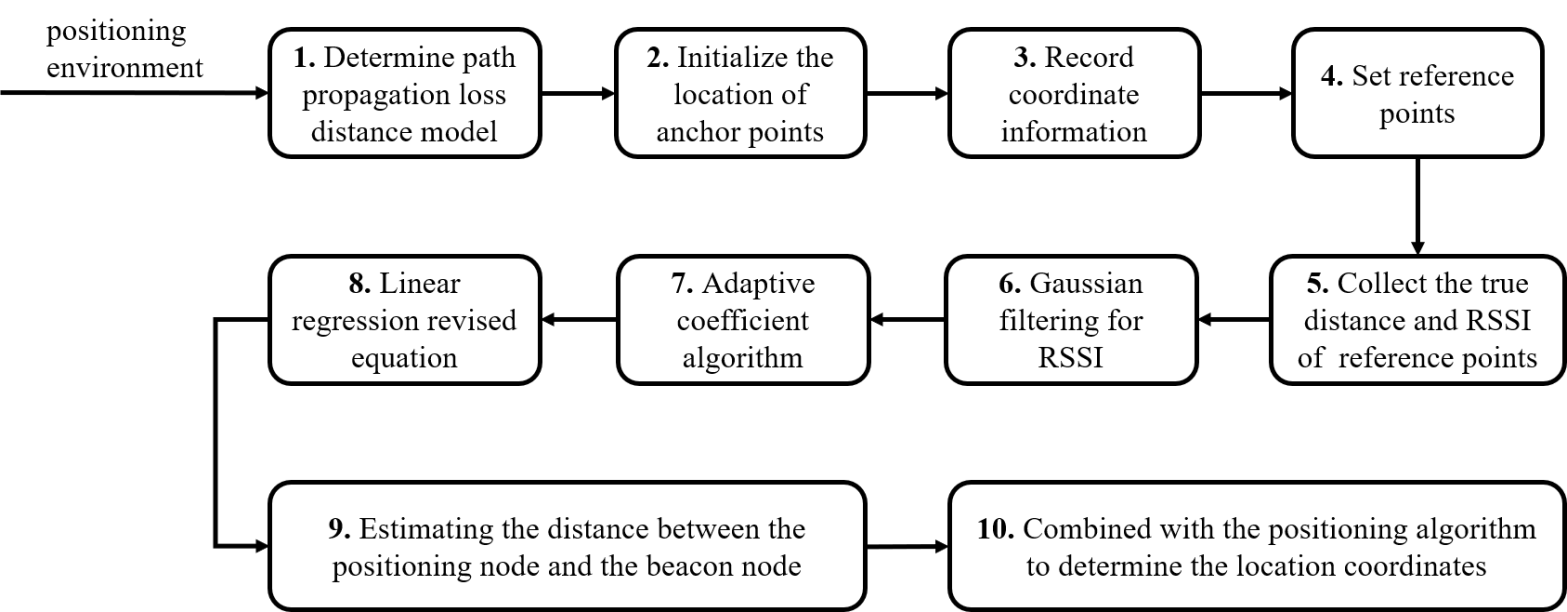
The localization process of indoor positioning.

Taking three reference points as an example, the indoor positioning steps based on the propagation model method are as follows. And the positioning process is shown in Figure 1.

(1) Determine the path propagation loss distance model according to the positioning environment.

(2) Initialize the position of anchor points and record their relative coordinate information.

(3) Set reference points in the positioning area and collect the real distance and RSSI values, and determine the model parameters in the positioning environment according to the information of the sample.

(4) Use the RSSI collected by to-be-positioned points and the obtained signal propagation loss model to estimate the distance between to-be-positioned points and anchor points.

(5) Using the estimated distances to three anchor points estimated by to-be-positioned points, combined with a positioning algorithm to determine the relative position coordinates of to-be-positioned points.

## 4. Experiments and Discussions

In this section, we first introduce the algorithm performance evaluation indicator and the experimental setup, followed by experimental results and discussions.

### 4.1. Evaluating Indicator

Due to the influence of hardware equipment, indoor communication environment, and other factors, there are certain errors in the measurement of various positioning parameters, resulting in errors in the final estimated target position coordinates. In this paper, the localization error is used to evaluate the localization accuracy of the model. The location error ERROR is generally expressed by the Euclidean distance between the estimated location and the true location

(20) $$ERROR=\sqrt{{\left(x_{0}-x_{d}\right)}^{2}-{\left(y_{0}-y_{d}\right)}^{2}}$$

where $\left(x_{0},y_{0}\right)$ represents the real coordinate location of the positioning user, and $\left(x_{i},y_{i}\right)$ represents the location coordinates estimated by the positioning algorithm. It should be noted that the coordinate position described in the process of indoor positioning in this paper is the relative coordinate in the experimental scene rather than the real geographic coordinate.

### 4.2. Experimental Setup

**Figure 2:**
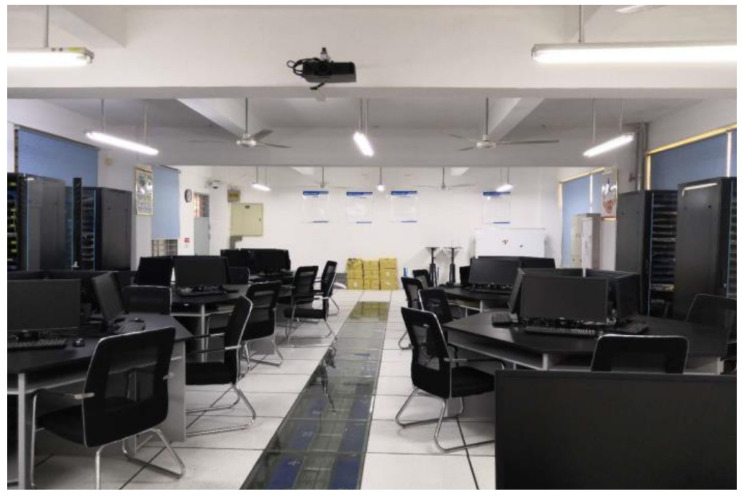
Experiment environment.

**Figure 3:**
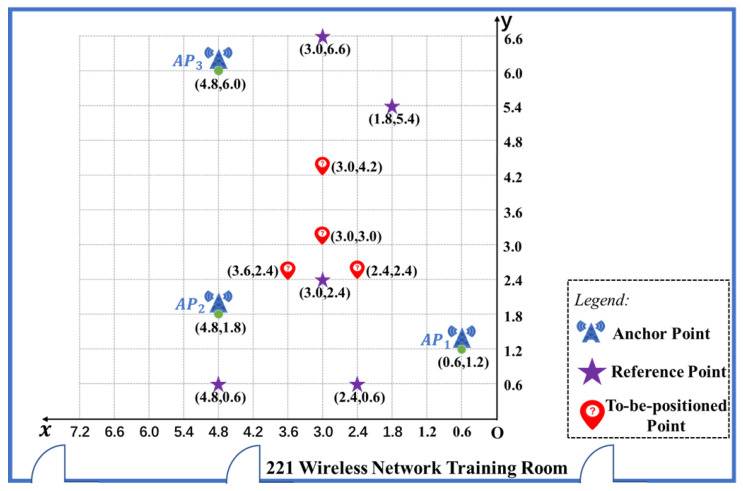
Experimental scene distribution map.

**Figure 4:**
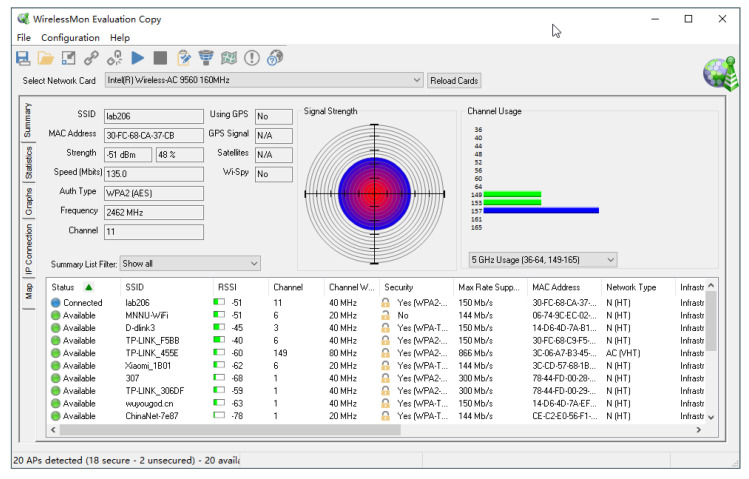
The signal acquisition interface.

The experimental environment in this paper was set in the wireless network training room 221 on the second floor of Li Zhi Building, Minnan Normal University. The real scene of the experimental environment is shown in Figure 2. In this experiment, three anchor points were set with the coordinates (0.6, 1.2), (4.8, 1.8), and (4.8, 6.0), respectively. At the same time, five reference points were set, and their coordinates are (3.0, 2.4), (3.0, 6.6), (1.8, 5.4), (2.4, 0.6), and (4.8, 0.6), respectively. There were also four to-be-positioned points. The coordinate unit is meter, abbreviated as *m*. The reference points are used to establish the propagation model, and the to-be-positioned points are used to verify the effect of models. Three signal transmitting devices TL-WR842N were placed at the three anchor points, respectively. TL-WR842N is a cheap and easily available small office & home office (SOHO) wireless router which is produced by TP-Link Corporation Limited. TL-WR842N adopts 802.11n wireless technology, and the wireless transmission rate is up to 300 M bps at the 2.4 GHz band. It also adopts 2 × 2 multiple-input & multiple-output (MIMO) technology with 2 external antennas, which can provide stable wireless connections and optimal coverage. For convenience, the three TL-WR842N devices were represented as $AP_{1}$, $AP_{2}$, and $AP_{3}$, respectively. The five reference points were represented as A,B,C,D, and *E*, respectively. The four to-be-positioned points were represented as p_a,p_b,p_c, and p_d, respectively. The distribution of anchor points, reference points, and to-be-positioned points is shown in Figure 3. The omni-directional antennas of TL-WR842N were all perpendicular to the ground. The service set identifiers (SSIDs) of the three TL-WR842N were named TP-LINK_F5B9, TP-LINK_CB37, and TP-LINK_A2F4, respectively. The transmission power of three TL-WR842N were all set as default power uniformly. To reduce the co-channel interference and adjacent frequency interference, the three TL-WR842N used wireless channels 1, 5, and 12, respectively. The Lenovo notebook computer with Realtek 8821AE Wireless LAN 802.11ac PCI-E Wireless network card was used as the receiver to collect the RSSI of the three signal transmitting devices TL-WR842N. The WirelessMON with version 4.0 was used as the RSSI signal acquisition software. Media access control (MAC) address, SSID, collection time, and RSSI of each AP were recorded respectively. The signal acquisition interface of WirelessMON is shown in Figure 4.

There are many objects in the laboratory that affect the transmission of Wi-Fi signals, such as desks, computers, switches, etc. We collected RSSI signals at five reference points and four to-be-positioned points according to the following rules. At each point of reference points and to-be-positioned points, for each anchor point, 60 RSSI signals were collected continuously at one time. To reduce the interference of multi-path effects and other factors, RSSI signals were collected twice in turn. Therefore, 1080 RSSI signals were obtained for each anchor point, and 3240 data were obtained for all three anchor points. The RSSI Gaussian filtering algorithm was used to process the collected RSSI signals, and then calculated the arithmetic mean value of the RSSI signal collected at each anchor point as the RSSI value of the anchor point collected at that point.

After processing the collected RSSI with the RSSI Gaussian filtering algorithm, the measurement data of reference points and to-be-positioned points are shown in Table 1 and Table 2.

In the Table 1 and Table 2, $d_{1}$, $d_{2}$, and $d_{3}$ represent the actual measured distances to $AP_{1}$, $AP_{2}$, and $AP_{3}$, respectively. $R(d_{1})$, $R(d_{2})$, and $R(d_{3})$, represent the mean values of the actual measured RSSI signals collected from $AP_{1}$, $AP_{2}$, and $AP_{3}$, respectively. $X(d_{1})$, $X(d_{2})$, and $X(d_{3})$ denote the actual measurement standard deviations of RSSI from $AP_{1}$, $AP_{2}$, and $AP_{3}$ obtained by continuous measurement, respectively.

### 4.3. Ranging Results of Classic LDPLM

By substituting the values in Table 1 into Equation (16), the coefficients $R_{0}\left(d_{0}\right)$ and *n* of the classic LDPLM can be obtained. Then the coefficients of each AP transmitter are obtained as shown in Table 3, and they are re-substituted into the model to observe and fit the current indoor environment.

The RSSI values of the measured data in Table 2 and the coefficients of the classic LDPLM in Table 3 are substituted into Equations (4). The calculated distance is obtained and compared with the actual measured distance. The results are shown in Table 4.

In Table 4, $d_{1}^{{}^{\prime }}$, $d_{2}^{{}^{\prime }}$, and $d_{3}^{{}^{\prime }}$ represent the solution distance of the model from the to-be-positioned point to $AP_{1}$, $AP_{2}$, and $AP_{3}$ respectively. $diff_{1}\left(d\right)$, $diff_{2}\left(d\right)$, and $diff_{3}\left(d\right)$ represent the absolute error between the calculated distance and the actual measured distance from each to-be-positioned point to $AP_{1}$, $AP_{2}$, and $AP_{3}$ respectively. S_diff is the sum of the error between the settlement distances of the model and the actual measurement distances from each to-be-positioned point to three APs, i.e., the sum of $diff_{1}\left(d\right)$, $diff_{2}\left(d\right)$, and $diff_{3}\left(d\right)$.

### 4.4. Ranging Results of Polynomial Fitting Ranging Model

By substituting the values in Table 1 into Equation (5), the coefficients *a*, *b*, and *c* can be obtained by the least square method. The coefficients of each TP-LINK transmitter are shown in Table 5. Then they are replaced with the polynomial fitting ranging model to observe and fit the current indoor environment.

The RSSI values of the measured data in Table 2 and the parameter estimation values of the polynomial fitting ranging model in Table 5 are substituted into Equation (5). The calculated distances can be obtained and compared with the actual measured distances. The results are shown in Table 6.

Because the ranging model based on the polynomial fitting will become a quadratic equation of one variable when solving the solution, there will be non-existent solutions or existing negative solutions, but the actual distance cannot be negative. To fully prove the accuracy of the adaptive calibration algorithm proposed in this paper and make the experimental results more convincing, the optimal value obtained by the polynomial fitting ranging model is retained in Table 6. That is, in the case of negative solutions, the negative values of the two distance solutions are eliminated and the positive solutions are selected. At the same time, in the case of two positive solutions, the solution closer to the actual distance is taken as the model solution. “-” in Table 6 indicates that the equation has no solution, that is, in some cases, the distance can not be calculated through the polynomial fitting ranging model.

### 4.5. Ranging Results of Adaptive Calibration Algorithm

Coefficients *a* and *b* can be obtained by substituting the values in Table 1 into Equation (19). The solved coefficients of each TP-LINK transmitter are shown in Table 7. And then they are brought back into the error correction algorithm to observe and fit the current indoor environment.

Substitute the RSSI values of the measured data in Table 2 and the solved coefficients of the error correction linear regression equation in Table 7 into Equation (17) to obtain the calculated distances, which are compared with the actual measurement distances. The results are shown in Table 8.

### 4.6. Comparison and Analysis of Models’ Ranging Results

Extract the last column of attribute value S_diff from Table 4, Table 6 and Table 8, which are respectively expressed as $S_{i}\_diff$, i=1,2,3, and get the result as shown in Table 9.

The p_a, p_b, p_c, p_d in Table 9 represent the to-be-positioned points. Because the coordinates of each to-be-positioned point are obtained by the joint action of three APs. Therefore, the smaller the $S_{i}\_diff$, the more accurate the ranging result of the to-be-positioned point, and the more helpful to improve the positioning accuracy. SUM is the sum of four to-be-positioned points $S_{i}\_diff$, i=1,2,3. It represents the sum of the error between the model calculated distance and the actual measured distance from all to-be-positioned points to three AP transmitters. The smaller the SUM, the better the positioning accuracy.

“-” in Table 9 indicates that the polynomial fitting ranging model fails to calculate the model distance in some cases. It can be seen from Table 9 that four to-be-positioned points *a*, *b*, *c*, and *d*, the $S_{3}\_diff$ obtained by the proposed model is smaller than $S_{1}\_diff$ and $S_{2}\_diff$ obtained by the former two models. This means that for a single to-be-positioned point, the ranging error obtained by the proposed model is smaller than that of the former two models. At the same time, it can be seen from the SUM value that the total ranging error of all to-be-positioned points of the proposed model is 3.56, which is less than 8.57 and 7.28 of the former two models, and the total ranging accuracy is improved by 58% and 51%, respectively.

### 4.7. Comparison and Analysis of Models’ Positioning Results

Based on the ranging results obtained from the above three ranging models, we calculated the specific coordinates of each model for to-be-positioned points by the least square positioning algorithm. The results are shown in Table 10. In Table 10, model 1, model 2, and model 3 represent the classic LDPLM model, the polynomial fitting ranging model, and the adaptive calibration model, respectively.

**Figure 5:**
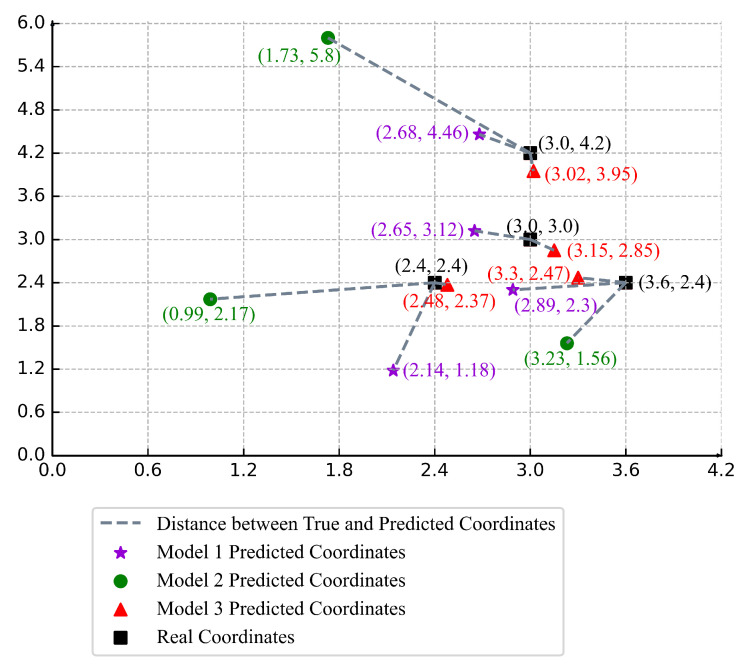
Comparison of model predictions and real coordinates.

**Figure 6:**
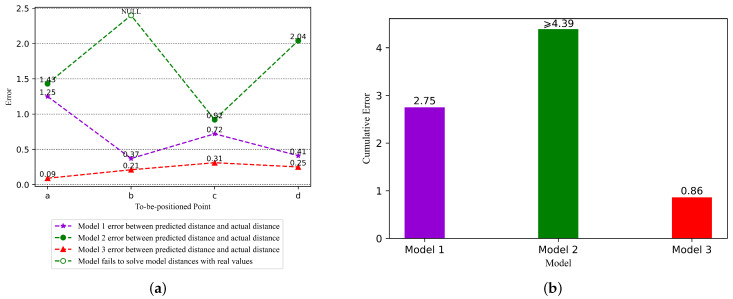
Comparison of positioning prediction errors of three models. (**a**) Prediction error distribution plots for the three models. (**b**) Cumulative errors for the three models.

To compare the model results more intuitively, we drew a comparison chart of the actual coordinates of to-be-positioned points and calculated coordinates of the three models, as shown in Figure 5. In Figure 5, “★” represents the calculated coordinates of the to-be-positioned points by model 1, “●” represents the calculated coordinates of the to-be-positioned points by model 2, “▲” represents the calculated coordinates of the to-be-positioned points by model 3, and “*■*” represents the actual coordinates of the to-be-positioned points. The connecting line between the actual coordinates of the to-be-positioned points and the model calculated coordinates of the to-be-positioned points represents the prediction error between them. The connecting line can be expressed by the Euclidean distance, that is, the Equation (20) represented in this paper. In other words, we used the Euclidean distance to evaluate the positioning prediction errors of the three models. The prediction errors of the three models at the four to-be-positioned points a,b,c, and *d* are shown in Figure 6a. And the cumulative prediction errors of the three models are shown in Figure 6b.

In Figure 6a, we used graphics with different shapes to represent different models, where “★” represents the prediction error of model 1, “●” represents the prediction error of model 2, and “▲” represents the prediction error of model 3. The prediction error of model 2 at the to-be-positioned point *b* is “NULL”. The reason is that model 2 cannot solve the distance at the to-be-positioned point *b* and thus cannot obtain the calculated coordinate.

From Figure 6a, it can be found that for each to-be-positioned point, the prediction error of the adaptive calibration model (model 3) proposed in this paper is the smallest, the classic LDPLM model (model 1) is the second, and the polynomial fitting ranging model (model 2) is the largest. From Figure 6b, it can be found that the cumulative prediction error of the adaptive calibration model (model 3) is also the smallest, the classic LDPLM model (model 1) is the second, and the polynomial fitting ranging model (model 2) is the largest. Compared with model 1 and model 2, the positioning cumulative prediction error of the proposed model is reduced by 69% and 80%, respectively.

## 5. Conclusions

In this paper, we propose the adaptive calibration algorithm based on LDPLM and RSSI for indoor ranging and positioning. The adaptive calibration algorithm consists of two parts: coefficient adaptive algorithm and error correction algorithm. The coefficient adaptive algorithm solves the problem that the coefficients of the classic LDPLM cannot be adjusted adaptively. While the error correction algorithm reduces the error caused by the coefficient adaptive algorithm. Furthermore, we design a simple but effective filtering algorithm based on Gaussian to process RSSI. The experimental results show that our work effectively improves the ranging accuracy and reduces the positioning prediction error and the positioning cumulative prediction error. Future works may further consider the real-time performance and computational complexity of the proposed system at a practical level. In addition, the adaptability of the proposed system in different wireless networks, such as the low-power internet of things (IoT), also needs further consideration.

## Figures and Tables

**Table 1:** Measurement data of reference points.

Reference Point	Coordinates	$R(d_{1})$ /dBm	$R(d_{2})$ /dBm	$R(d_{3})$ /dBm	$d_{1}$ /m	$d_{2}$ /m	$d_{3}$ /m	$X(d_{1})$ /dBm	$X(d_{2})$ /dBm	$X(d_{3})$ /dBm
*A*	(3.0, 2.4)	−48.45	−37.17	−57.50	2.68	1.90	4.03	2.50	3.01	1.37
*B*	(3.0, 6.6)	−56.08	−55.53	−49.90	5.91	5.13	1.90	2.04	1.89	1.54
*C*	(1.8, 5.4)	−60.10	−57.63	−41.30	4.37	4.69	3.06	2.45	1.64	0.94
*D*	(2.4, 0.6)	−44.25	−46.20	−54.63	1.90	2.68	5.91	2.94	2.19	1.04
*E*	(4.8, 0.6)	−49.25	−46.87	−59.68	4.24	1.20	5.40	3.10	2.22	1.39

**Table 2:** Measured data of to-be-positioned points.

To-Be-Positioned Point	Coordinates	$R(d_{1})$ /dBm	$R(d_{2})$ /dBm	$R(d_{3})$ /dBm	$d_{1}$ /m	$d_{2}$ /m	$d_{3}$ /m
p_a	(2.4, 2.4)	−45.50	−51.00	−57.43	2.16	2.47	4.33
p_b	(3.0, 3.0)	−45.75	−41.88	−51.02	3.00	2.16	3.50
p_c	(3.6, 2.4)	−48.07	−43.32	−54.13	3.23	1.34	3.79
p_d	(3.0, 4.2)	−53.33	−50.77	−45.88	3.84	3.00	2.55

**Table 3:** Parameter estimation of Classic LDPLM.

Transmitter	$R_{0}(d_{0}$)/dBm	*n*
$AP_{1}$	−40.51	2.50
$AP_{2}$	−41.48	2.16
$AP_{3}$	−41.62	2.13

**Table 4:** The classic LDPLM ranging results and errors (m).

To-Be-Positioned Point	$d_{1}^{{}^{\prime }}$	$d_{2}^{{}^{\prime }}$	$d_{3}^{{}^{\prime }}$	${\mathrm{diff}}_{1}\left(d\right)$	${\mathrm{diff}}_{2}\left(d\right)$	${\mathrm{diff}}_{3}\left(d\right)$	S_diff
p_a	1.58	2.76	5.52	0.58	0.29	1.19	2.06
p_b	1.62	1.04	2.76	1.38	1.12	0.74	3.24
p_c	2.01	1.23	3.87	1.22	0.11	0.08	1.41
p_d	3.26	2.69	1.58	0.58	0.31	0.97	1.86

**Table 5:** Parameter estimation of polynomial fitting model.

Transmitter	*a*	*b*	*c*
$AP_{1}$	−29.16	−9.40	0.81
$AP_{2}$	−48.49	5.66	−1.47
$AP_{3}$	−43.56	−1.46	−0.17

**Table 6:** Polynomial fitting ranging model ranging results and errors (m).

To-Be-Positioned Point	$d_{1}^{{}^{\prime }}$	$d_{2}^{{}^{\prime }}$	$d_{3}^{{}^{\prime }}$	${\mathrm{diff}}_{1}\left(d\right)$	${\mathrm{diff}}_{2}\left(d\right)$	${\mathrm{diff}}_{3}\left(d\right)$	S_diff
p_a	2.13	4.25	5.17	0.03	1.78	1.38	3.19
p_b	2.17	-	3.60	0.83	-	0.10	-
p_c	2.59	1.49	4.68	0.64	0.15	0.89	1.68
p_d	3.85	4.22	1.37	0.01	1.22	1.18	2.41

**Table 7:** The solved coefficients of error correction linear regression equation.

Transmitter	*a*	*b*
$AP_{1}$	2.08	0.54
$AP_{2}$	1.06	0.73
$AP_{3}$	2.24	0.45

**Table 8:** The ranging results and errors of the adaptive calibration algorithm (m).

To-Be-Positioned Point	$d_{1}^{{}^{\prime }}$	$d_{2}^{{}^{\prime }}$	$d_{3}^{{}^{\prime }}$	${\mathrm{diff}}_{1}\left(d\right)$	${\mathrm{diff}}_{2}\left(d\right)$	${\mathrm{diff}}_{3}\left(d\right)$	S_diff
p_a	2.93	3.07	4.72	0.77	0.60	0.39	1.76
p_b	2.95	1.82	3.48	0.05	0.34	0.02	0.41
p_c	3.17	1.96	3.98	0.06	0.62	0.19	0.87
p_d	3.84	3.02	2.95	0.00	0.02	0.40	0.42

**Table 9:** Error comparison of three ranging models (m).

	p_a	p_b	p_c	p_d	SUM
$S_{1}\_diff$	2.06	3.24	1.41	1.86	8.57
$S_{2}\_diff$	3.19	-	1.68	2.41	≥7.28
$S_{3}\_diff$	1.76	0.41	0.87	0.42	3.56

**Table 10:** The estimated coordinates of the three models (m).

To-Be-Positioned Point	Real Coordinates	Model 1	Model 2	Model 3
p_a	(2.40, 2.40)	(2.14, 1.18)	(0.99, 2.17)	(2.48, 2.37)
p_b	(3.00, 3.00)	(2.65, 3.12)	-	(3.15, 2.85)
p_c	(3.60, 2.40)	(2.89, 2.30)	(3.23, 1.56)	(3.30, 2.47)
p_d	(3.00, 4.20)	(2.68, 4.46)	(1.73, 5.80)	(3.02, 3.95)

## Data Availability

Not applicable.
